# The Hematological Complications of Alcoholism

**Published:** 1997

**Authors:** Harold S. Ballard

**Affiliations:** Harold S. Ballard, M.D., is associate chief of hematology and oncology at the New York Department of Veterans Affairs Medical Center, New York, New York

**Keywords:** adverse drug effect, AODE (alcohol and other drug effects), blood function, cell growth and differentiation, erythrocytes, leukocytes, platelets, plasma proteins, bone marrow, anemia, blood coagulation, thrombocytopenia, fibrinolysis, macrophage, monocyte, stroke, bacterial disease, literature review

## Abstract

Alcohol has numerous adverse effects on the various types of blood cells and their functions. For example, heavy alcohol consumption can cause generalized suppression of blood cell production and the production of structurally abnormal blood cell precursors that cannot mature into functional cells. Alcoholics frequently have defective red blood cells that are destroyed prematurely, possibly resulting in anemia. Alcohol also interferes with the production and function of white blood cells, especially those that defend the body against invading bacteria. Consequently, alcoholics frequently suffer from bacterial infections. Finally, alcohol adversely affects the platelets and other components of the blood-clotting system. Heavy alcohol consumption thus may increase the drinker’s risk of suffering a stroke.

People who abuse alcohol^1^ are at risk for numerous alcohol-related medical complications, including those affecting the blood (i.e., the blood cells as well as proteins present in the blood plasma) and the bone marrow, where the blood cells are produced. (For more information on the blood’s composition and on the various types of blood cells and their production, see sidebar, pp. 50–51.) Alcohol’s adverse effects on the blood-building, or hematopoietic, system are both direct and indirect. The direct consequences of excessive alcohol consumption include toxic effects on the bone marrow; the blood cell precursors; and the mature red blood cells (RBC’s), white blood cells (WBC’s), and platelets. Alcohol’s indirect effects include nutritional deficiencies that impair the production and function of various blood cells.

**Blood Development and Composition**

Blood vessels reach every organ and tissue in the body, indicating that the blood and the integrity of the blood vessels are essential to maintaining the body’s health and functioning. The blood’s most important functions include transporting substances, such as oxygen, nutrients, waste products to be excreted, and chemical messengers; defending the body against foreign organisms and substances, such as bacteria, viruses, and fungi; and repairing injured blood vessels.

Blood cells make up about 45 percent of the blood volume; the remaining 55 percent consists of a watery liquid called plasma. In addition to water, plasma contains minerals; nutrients; regulatory substances, such as homones; gases, such as oxygen and carbon dioxide; and proteins. These proteins include those involved in blood clotting as well as immune proteins (i.e., antibodies or immunoglobulins).

***Blood Cells***

Blood cells fall into three major categories, each of which has specific functions, as follows:

- Red blood cells (RBC’s), also called erythrocytes, transport oxygen from the lungs to all the cells in the body and carry carbon dioxide from the cells back to the lungs.
- White blood cells (WBC’s), or leukocytes, engage in the body’s defense against foreign microorganisms or toxic substances as well as mediate the immune response.
- Platelets help maintain the integrity of the blood vessels by stimulating blood clotting (i.e., coagulation) after an injury and thereby stopping the bleeding.

**Figure:**
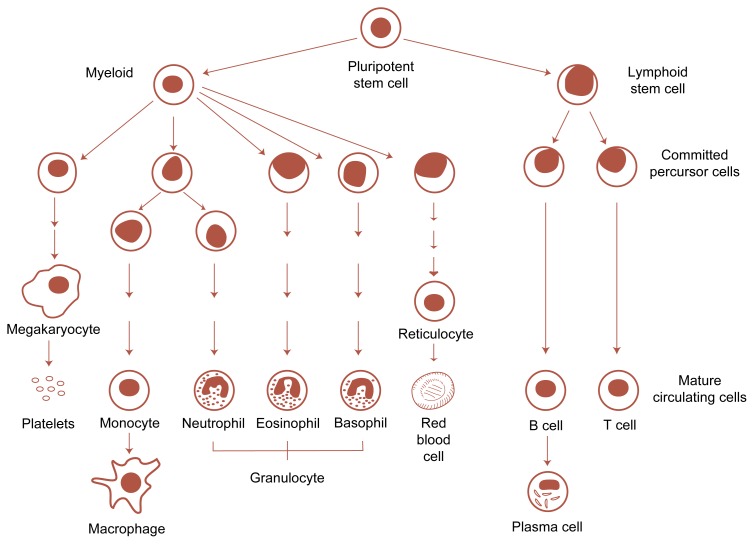
Blood cell development. All types of circulating blood cells develop from a pluripotent stem cell. Under the influence of certain proteins (i.e., growth factors), this stem cell multiplies and differentiates into increasingly committed precursor cells. Through several intermediate stages, these precursors differentiate further and develop into the mature cells circulating in the blood or residing in the tissues.

The WBC’s are subdivided further into three groups—granulocytes, monocytes and macrophages, and lymphocytes—each with specific functions. Granulocytes recognize and eliminate microorganisms, especially bacteria, by ingesting them. Several types of granulocytes exist. Cells of the most common type are called neutrophils. Monocytes (which circulate in the blood) and macrophages (monocytes that have entered the tissues) also ingest and destroy foreign organisms and substances. In addition, these cells display foreign proteins and other molecules (i.e., antigens) on their surface and thereby help activate the body’s immune response. Lymphocytes, which mediate and regulate the immune response, consist of two main groups: T cells and B cells. T cells secrete messenger substances (i.e., cytokines) that activate and regulate the immune response. T cells also kill cells displaying foreign antigens on their surface (e.g., virus-infected or transplanted cells). B cells produce and secrete antibodies that can bind to foreign antigens. This binding often is required to initiate the elimination of foreign microorganisms.

***Hematopoiesis***

Although the different blood cells have distinct structures and functions, they are all produced at the same site, the bone marrow, in a complex process called hematopoiesis. Hematopoiesis involves the multiplication (i.e., proliferation) of precursor cells, their specialization (i.e., differentiation) into cells with a specific function (e.g., oxygen transport or antibody secretion), and their maturation into functional cells that eventually circulate in the blood. The production of all types of blood cells begins with undifferentiated precursor cells—so-called pluripotent stem cells—that can develop into whichever cell type is needed at that time. Through a series of tightly regulated intermediary stages, these stem cells multiply, differentiate, and mature into hundreds of thousands of functional RBC’s, WBC’s, or platelets, which then can be released into the bloodstream (see figure).

***Detection of Blood Disorders***

To detect blood disorders, physicians frequently examine small blood samples (known as blood smears) under a microscope and assess the appearance, size, and number of the various blood cells. Each type of blood cell has a characteristic appearance that allows its identification in blood samples. Moreover, the proportion of the different cell types in the blood is relatively constant. Consequently, physicians can diagnose many blood disorders based on changes in the appearance or proportion of certain blood cells. For example, stomatocytosis (an RBC disorder; see main text) is characterized by abnormal, mouth-shaped RBC’s.

Another way to identify blood disorders is to perform a complete blood count (CBC), in which a machine counts all the cells within a blood sample. In addition, these machines can determine several other parameters of blood cells, such as their average size, which may be diagnostic for certain disorders. For example, an increase in the average RBC volume (i.e., the mean corpuscular volume [MCV]) is characteristic for a certain type of anemia.

Many blood disorders result from impaired or abnormal production of blood cells. These disorders can be diagnosed by microscopic analysis of bone marrow samples;^1^ This type of diagnosis allows the physician to determine the overall number of cells in the bone marrow as well as the proportion of abnormal cells. Moreover, because each of the intermediary precursors of the various blood cell types has a characteristic appearance that can be discerned during microscopic examination, physicians can identify certain blood disorders based on the number and type of specific precursors in the marrow. For example, different types of leukemia are characterized by the accumulation in the bone marrow of WBC precursors at specific developmental stages.

—*Susanne Hiller-Sturmhöfel*

*Susanne Hiller-Sturmhöfel is a science editor of* Alcohol Health & Research World.1Bone marrow samples can be obtained by withdrawing tissue from the bone’s interior with a needle or by removing a small “core” of marrow.

These direct and indirect effects of alcohol can result in serious medical problems for the drinker. For example, anemia^2^ resulting from diminished RBC production and impaired RBC metabolism and function can cause fatigue, shortness of breath, lightheadedness, and even reduced mental capacity and abnormal heartbeats. A decrease in the number and function of WBC’s increases the drinker’s risk of serious infection, and impaired platelet production and function interfere with blood clotting, leading to symptoms ranging from a simple nosebleed to bleeding in the brain (i.e., hemorrhagic stroke). Finally, alcohol-induced abnormalities in the plasma proteins that are required for blood clotting can lead to the formation of blood clots (i.e., thrombosis).

This article summarizes current information on the consequences of excessive alcohol consumption on the bone marrow and on the production and function of RBC’s, WBC’s, platelets, and plasma proteins.

## Alcohol’s Effects on the Bone Marrow and on RBC Production

Alcohol is the most commonly used drug whose consequences include the suppression of blood cell production, or hematopoiesis. Because its toxic effects are dose dependent, however, significantly impaired hematopoiesis usually occurs only in people with severe alcoholism, who also may suffer from nutritional deficiencies of folic acid and other vitamins that play a role in blood cell development. Chronic excessive alcohol ingestion reduces the number of blood cell precursors in the bone marrow and causes characteristic structural abnormalities in these cells, resulting in fewer-than-normal or nonfunctional mature blood cells. As a result, alcoholics may suffer from moderate anemia, characterized by enlarged, structurally abnormal RBC’s; mildly reduced numbers of WBC’s, especially of neutrophils; and moderately to severely reduced numbers of platelets. Although this generalized reduction in blood cell numbers (i.e., pancytopenia) usually is not progressive or fatal and is reversible with abstinence, complex aberrations of hematopoiesis can develop over time that may cause death.

Many bone marrow abnormalities occurring in severe alcoholics affect the RBC precursor cells. These abnormalities most prominently include precursors containing fluid-filled cavities (i.e., vacuoles) or characteristic iron deposits.

### Development of Vacuoles in RBC Precursors

The most striking indication of alcohol’s toxic effects on bone marrow cells is the appearance of numerous large vacuoles in early RBC precursor cells. It is unknown whether these vacuoles affect the cell’s function and thus the drinker’s health; however, their appearance generally is considered an indicator of excessive alcohol consumption.^3^ The vacuoles usually appear in the pronormoblasts 5 to 7 days following the initiation of heavy alcohol consumption. Moreover, the vacuoles on average disappear after 3 to 7 days of abstinence, although in some patients they persist for up to 2 weeks.

To a lesser extent, vacuoles also develop in the granulocyte precursors of alcoholics. This finding is not specifically alcohol related, however, because other events that interfere with WBC production (e.g., infections) may induce similar structural changes in the granulocyte precursors.

The precise mechanism underlying vacuole development in blood cell precursors currently is unknown. Microscopic analyses of early blood cell precursors grown in tissue culture suggest that when the cells are exposed to a wide range of alcohol concentrations, the membrane surrounding each cell is damaged. These alterations in membrane structure may play an influential role in vacuole formation.

### Sideroblastic Anemia

One component of RBC’s is hemoglobin, an iron-containing substance that is essential for oxygen transport. Sometimes, however, the iron is not incorporated properly into the hemoglobin molecules. Instead, it is converted into a storage form called ferritin, which can accumulate in RBC precursors, often forming granules that encircle the cell’s nucleus. These ferritin-containing cells, which are called ringed sideroblasts, cannot mature further into functional RBC’s. As a result, the number of RBC’s in the blood declines and patients develop anemia. Many patients also have some circulating RBC’s that contain ferritin granules called Pappenheimer bodies. The presence of these cells in the blood serves as an indicator of sideroblastic anemia and can prompt the physician to perform a bone marrow examination to confirm the diagnosis.

Sideroblastic anemia is a common complication in severe alcoholics: Approximately one-third of these patients contain ringed sideroblasts in their bone marrow. Alcohol may cause sideroblastic anemia by interfering with the activity of an enzyme that mediates a critical step in hemoglobin synthesis. (For information on other effects of alcohol on iron metabolism, see box, p. 43) Abstinence can reverse this effect: The ringed sideroblasts generally disappear from the bone marrow within 5 to 10 days, and RBC production resumes. In fact, excess numbers of young RBC’s called reticulocytes can accumulate temporarily in the blood, indicating higher-than-normal RBC production.

**Alcohol’s Effects on Iron Metabolism**

In addition to interfering with the proper absorption of iron into the hemoglobin molecules of red blood cells (RBC’s), alcohol use can lead to either iron deficiency or excessively high levels of iron in the body. Because iron is essential to RBC functioning, iron deficiency, which is commonly caused by excessive blood loss, can result in anemia. In many alcoholic patients, blood loss and subsequent iron deficiency are caused by gastrointestinal bleeding. Iron deficiency in alcoholics often is difficult to diagnose, however, because it may be masked by symptoms of other nutritional deficiencies (e.g., folic acid deficiency) or by coexisting liver disease and other alcohol-related inflammatory conditions. For an accurate diagnosis, the physician must therefore exclude folic acid deficiency and evaluate the patient’s iron stores in the bone marrow.

Conversely, alcohol abuse can increase iron levels in the body. For example, iron absorption from the food in the gastrointestinal tract may be elevated in alcoholics. Iron levels also can rise from excessive ingestion of iron-containing alcoholic beverages, such as red wine. The increased iron levels can cause hemochromatosis, a condition characterized by the formation of iron deposits throughout the body (e.g., in the liver, pancreas, heart, joints, and gonads). Moreover, patients whose chronic alcohol consumption and hemochromatosis have led to liver cirrhosis are at increased risk for liver cancer.

### Megaloblastic Anemia

Blood cell precursors require folic acid and other B vitamins for their continued production. Under conditions of folic acid deficiency, precursor cells cannot divide properly and large immature and nonfunctional cells (i.e., megaloblasts) accumulate in the bone marrow as well as in the bloodstream. This impaired hematopoiesis affects mainly RBC’s, but also WBC’s and platelets. The resulting deficiency in RBC’s, WBC’s, and platelets (i.e., pancytopenia) has numerous adverse consequences for the patient, including weakness and pallor from anemia, infections resulting from reduced neutrophil numbers, and bleeding as a result of the lack of platelets.

Megaloblasts occur frequently in the bone marrow of alcoholics; they are particularly common among alcoholics with symptoms of anemia, affecting up to one-third of these patients. These alcoholics generally also have reduced folic acid levels in their RBC’s. The most common cause of this deficiency is a diet poor in folic acid, a frequent complication in alcoholics, who often have poor nutritional habits. In addition, alcohol ingestion itself may accelerate the development of folic acid deficiency by altering the absorption of folic acid from food.

## Alcohol-Related RBC Disorders

**Figure 1:**
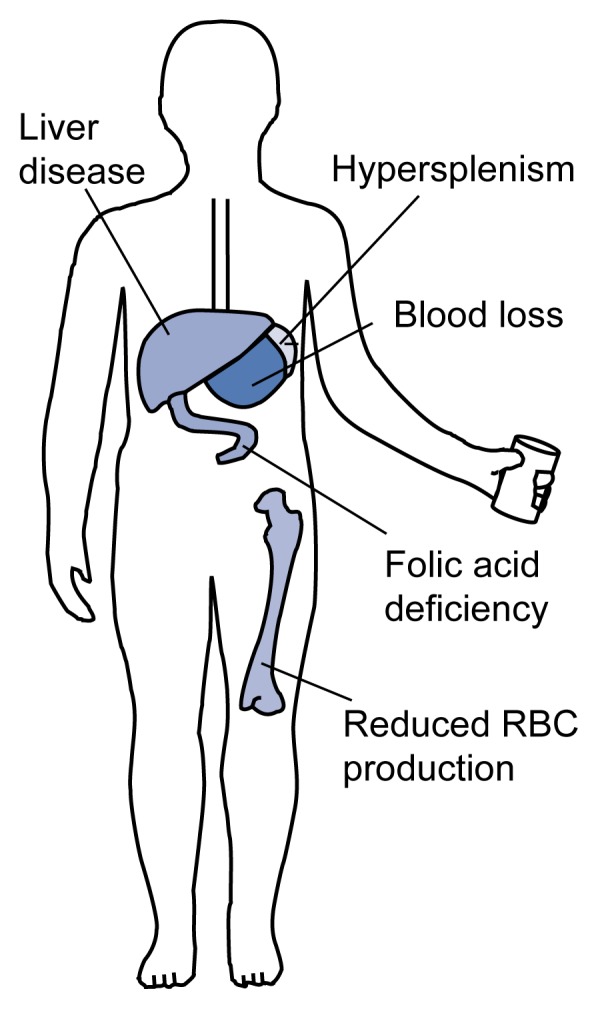
Causes of anemia in alcoholics. Alcohol, as well as alcohol-induced cirrhosis, leads to decreased red blood cell (RBC) production. Hypersplenism, a condition characterized by an enlarged spleen and deficiency of one or more blood cell types, can induce premature RBC destruction. Blood loss occurs primarily in the gastrointestinal tract (e.g., at the sites of peptic ulcers) and is increased in patients with reduced platelet numbers. Folic acid deficiency impairs RBC production and results from decreased ingestion, decreased absorption, and abnormal metabolism of folic acid. SOURCE: Adapted with permission from Cornwell, G.G., III. *Hematologic Complications of Alcohol. Unit 3*. Developed by the Project Cork Institute at Dartmouth Medical School. Timonium, MD: Milner-Fenwick, 1981.

Alcohol-related abnormalities in RBC production manifest themselves not only in the bone marrow but also through the presence of defective RBC’s in the blood. For example, grossly enlarged RBC’s can occur in the blood—a condition called macrocytosis—as well as oddly shaped RBC’s that are subject to premature or accelerated destruction (i.e., hemolysis) because of their structural abnormalities. As a result, alcoholics frequently are diagnosed with anemia (figure 1).

### Macrocytosis

The routine examination of blood samples from alcoholic and nonalcoholic patients using automated blood cell counters has resulted in the identification of many people in whom the average size of individual RBC’s—the mean corpuscular volume (MCV)—is significantly larger than normal. However, an increased MCV does not automatically lead to a diagnosis of macrocytosis. For example, cells with an increased MCV can be found in patients with folic acid or vitamin B_12_ deficiency (as in the case of megaloblastic anemia) or with chronic liver disease. Moreover, the presence of enlarged RBC’s in the blood can be indicative of a variety of disorders in addition to alcoholism, including different kinds of anemia and a dysfunction of the thyroid gland. To establish a diagnosis of macrocytosis, the physician must examine the blood cells under a microscope to identify structural features characteristic for each disorder. Thus, the enlarged RBC’s in patients with macrocytosis generally are uniformly round, in contrast to the more oval cells characteristic of megaloblastic anemia. In addition, a diagnosis of macrocytosis resulting from alcohol requires that the physician investigate all potential causes of RBC enlargement, including the patient’s alcohol-consumption history. (For more information on the use of the MCV and other blood-based variables as markers of alcohol consumption, see sidebar, p. 48–49.)

**Hematogical Markers of Alcoholism**

An important focus of alcohol research is the search for biological markers that could be used in simple screening tests to identify people who are at risk for alcoholism or who already are chronic heavy drinkers. Two categories of biological markers exist: state markers, which reflect a person’s alcohol consumption, and trait markers, which indicate a predisposition for alcoholism.

State markers fall into two main groups: screening markers and relapse markers. Screening markers, which detect chronic alcohol consumption, could complement information obtained from patients in the course of taking their medical history. This physical information could provide important diagnostic clues because, as clinical observations suggest, many people do not accurately report their level of alcohol consumption. Thus, screening markers could be useful in the early identification of alcoholism, especially in patients who consume alcohol in amounts that do not lead to acute medical problems but that could have long-term behavioral or medical consequences. In contrast, relapse markers, which are sensitive to acute alcohol consumption, could play an important role in monitoring recovering alcoholics and other heavy drinkers. State markers that would permit the identification of heavy drinkers even when alcohol is no longer present in the blood would be particularly valuable diagnostic tools.

Trait markers could help identify people at risk for alcoholism who could benefit most from early, targeted prevention and intervention approaches. These high-risk populations most prominently include first-degree relatives of alcoholics. Trait markers also could provide important research tools for evaluating the genetic and environmental factors that may predispose a person to alcoholism.

***State Markers***

Chronic ingestion of large quantities of alcohol alters many physiological and biological processes and compounds, including several blood-related (i.e., hematological) variables. Because blood samples are relatively easy to obtain, structural and functional changes in circulating blood cells and plasma proteins potentially can form the basis of laboratory tests for screening, diagnosing, and monitoring alcoholism. Two hematological state markers commonly used for these purposes are the presence of carbohydrate-deficient transferrin (CDT) in the blood and an increase in the size of red blood cells (RBC’s), as measured by the mean corpuscular volume (MCV).

***Carbohydrate-Deficient Transferrin***. CDT is one of the newest—and perhaps the most promising—of the hematological state markers. Transferrin is an iron-containing protein in the plasma that transports iron, which is stored at various sites in the body, to the developing RBC’s in the bone marrow for incorporation into hemoglobin. Transferrin molecules in the blood usually contain several carbohydrate components. In chronic heavy drinkers, however, the number of carbohydrate components in each transferrin molecule is reduced, resulting in CDT. The mechanism underlying this alteration still is unclear.

Because elevated CDT levels in the blood appear to be a specific consequence of excessive alcohol consumption, a recent study investigated the utility of repeatedly monitoring serum CDT to detect relapse among recovering alcoholics. The study found that in most of the subjects who relapsed, the elevation of CDT levels preceded self-reported alcohol consumption by at least 28 days. These findings suggest that repeated testing of alcoholic patients for CDT permits early relapse detection and thus may lead to early intervention. Early intervention, in turn, may decrease the need to rehospitalize patients for alcohol withdrawal and prevent some of the complications associated with sustained excessive drinking.

***Mean Corpuscular Volume***. The MCV is elevated in approximately 50 to 60 percent of people who chronically ingest excessive alcohol quantities. With the advent of automated instruments that determine the MCV during routine blood counts, physicians and other health care providers frequently detect elevated MCV’s in patients who are well nourished and who have no obvious disorders to explain this finding. In these patients, a moderately increased MCV may be a clue to unsuspected alcoholism. Analysis of blood smears can support this diagnosis: In patients with an alcohol-related increase in MCV, the enlarged RBC’s are round and of uniform size. Conversely, in patients with certain types of anemia that result in an increased MCV, the RBC’s typically are oval and of variable size. Because the MCV usually returns to normal within 2 to 4 months of abstinence, the increase in RBC size apparently is a direct effect of alcohol on RBC production.

***Trait Markers***

Researchers have proposed numerous genetic and genetically determined biochemical characteristics that might potentially serve as trait markers of alcoholism. Because of the easy availability of blood samples, many research efforts have focused on biochemical markers that can be found in circulating blood platelets. These studies have identified two enzymes that appear to be viable markers of alcoholism and whose activities can be measured in isolated platelets: monoamine oxidase (MAO) and adenylyl cyclase (AC).

***Monoamine Oxidase***. MAO is an enzyme that breaks down certain neurotransmitters (e.g., dopamine and serotonin) that have been implicated in mediating various phenomena related to the risk of developing alcoholism (e.g., tolerance to alcohol’s effects). Although MAO acts primarily in the brain, platelets also contain the enzyme. MAO activity levels are genetically determined, and many studies have demonstrated that people with a certain alcoholism subtype or with particular psychiatric disorders (e.g., schizophrenia and mood disorders) exhibit abnormally low MAO activity levels. In fact, low MAO activity in the platelets and other tissues of certain alcoholics is the most replicated biological finding in genetic alcoholism research. The available data also suggest that low MAO activity in the platelets predicts a risk for alcoholism in relatives of a certain type of alcoholics. This alcoholism subtype is characterized by an early age of onset of alcohol-related problems, frequent social and legal consequences of drinking, and a strong genetic predisposition.

***Adenylyl Cyclase***. AC is an enzyme that plays a role in the transmission of signals from a cell’s exterior to its interior; the enzyme’s levels in the body are genetically determined. Several studies have found that AC levels in the platelets as well as in some white blood cells are frequently reduced in alcoholics compared with nonalcoholics, even after long periods of abstinence. Because a single gene appears to determine the level of platelet AC activity, it is likely that low platelet AC activity is an inherited trait in many alcoholics and therefore could be used as a trait marker. Recent studies indicate, however, that the gene responsible for low AC levels does not actually cause alcoholism, but may increase the risk of developing the disease.

—*Harold S. Ballard*

BibliographyAnthenelliRMTabakoffBThe search for biochemical markersAlcohol Health & Research World1931761811995PMC687576131798085AntonRFThe use of carbohydrate deficient transferrin during treatment and follow-upAlcoholism: Clinical and Experimental Research20854A56A199610.1111/j.1530-0277.1996.tb01746.x8947235WhelanGBiological markers of alcoholismAustralian Journal of Medicine22209212199210.1111/j.1445-5994.1992.tb02815.x1382408

People who drink excessive amounts of alcohol can develop macrocytosis even in the absence of other factors associated with RBC enlargement, such as alcoholic liver disease or folic acid deficiency. In fact, alcohol abuse is the disorder most commonly associated with macrocytosis: Up to 80 percent of men and 46 percent of women with macrocytosis have been found to be alcoholics. The precise mechanism underlying macrocytosis still is unknown. However, alcohol appears to interfere directly with RBC development, because the macrocytes disappear within 2 to 4 months of abstinence.

### Hemolytic Anemia

Hemolysis can be an underlying cause of anemia, and several types of hemolytic anemia may be caused by chronic heavy alcohol consumption. Two of these disorders are characterized by the presence of malformed RBC’s—stomatocytes and spur cells—whereas one alcohol-related hemolytic anemia is caused by reduced phosphate levels in the blood (i.e., hypophosphatemia). Diagnosing hemolysis in alcoholic patients is not easy, because these patients frequently exhibit confounding conditions, such as alcohol withdrawal, abnormal folic acid levels, bleeding, or an enlarged spleen.

#### Stomatocyte Hemolysis

**Figure 2:**
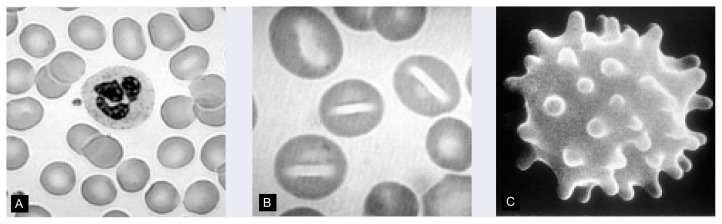
Alcohol-induced structural abnormalities in red blood cell (RBC) structure. (A) Normal RBC’s have a characteristic disclike shape; the cell in the center is a neutrophil. (B) Stomatocytes have a defect in their membranes that causes them to assume a mouth-, or stoma-, like shape when viewed under a microscope. (C) Spur cells are characterized by spikelike protrusions that result from the assimilation of excess cholesterol into the cell’s membrane. SOURCES: Images A and B are used with permission from the American Society of Hemotology Slide Bank. Image C is used with permission from Cornwell, G.G., III. *Hematologic Complications of Alcohol. Unit 3*. Developed by the Project Cork Institute at Dartmouth Medical School. Timinonium, MD: Milner-Fenwick, 1981.

Stomatocytes are RBC’s with a defect in their membranes that causes the cells to assume a mouth-, or stoma-, like shape when examined under a microscope (figure 2). Stomatocytes have a shortened life span because they become trapped in the small capillaries of the spleen and are subsequently destroyed. In healthy people, stomatocytes account for less than 5 percent of the RBC’s, whereas their number can be significantly higher in alcoholics. In fact, more than 25 percent of alcoholics exhibit an increased proportion of stomatocytes in the blood (i.e., stomatocytosis).

The exact mechanism by which alcohol causes the formation of stomatocytes still is unclear. Alcohol-related liver disease may play a role in the development of stomatocyte hemolysis, because all four of the binge-drinking alcoholics in whom stomatocytosis originally was identified also had some evidence of liver dysfunction. Alternatively, alcohol may directly affect the RBC’s. This hypothesis is supported by the observation that in the four original patients, the stomatocytes disappeared during abstinence, but reappeared when alcohol consumption was resumed.

#### Spur-Cell Hemolysis

Spur cells are distorted RBC’s that are characterized by spikelike protrusions of their cell membrane (figure 2). These spurs are caused by the incorporation of excess amounts of cholesterol into the cell membrane, resulting in an increase of the cell’s surface area without a corresponding increase in cell volume. Modestly elevated membrane cholesterol levels result in a flattened RBC shape, whereas larger increments of cholesterol cause the membrane to be thrown up into spikes. Spur cells may be prematurely eliminated in the spleen.

Spur-cell hemolysis occurs in about 3 percent of alcoholics with advanced liver disease, causing anemia that progresses relentlessly and is eventually fatal. Clinicians have tried unsuccessfully to treat the disorder using various agents with cholesterol-lowering properties. Consequently, surgical removal of the spleen is the only treatment capable of slowing the hemolytic process. Most alcoholic patients with spur-cell hemolysis, however, are not acceptable candidates for major abdominal surgery, because their coexisting advanced liver disease increases their risk of bleeding. Moreover, the procedure may precipitate liver failure.

#### Hypophosphatemia

Although hypophosphatemia-induced hemolysis is rare, its most common cause is alcoholism, especially during the withdrawal phase. Phosphate is an essential component of adenosine triphosphate (ATP), a compound that provides energy for many cellular processes. Alcohol causes phosphate to be excreted with the urine. Profound hypophosphatemia may cause the phosphate and ATP levels in the RBC’s to decline substantially. This depletion of the store of ATP in the RBC’s leads to increased rigidity of the RBC membranes, eventually damaging the cells. These damaged cells are prematurely destroyed in the spleen, and the patient may develop acute hemolytic anemia.

## Alcohol’s Effect on WBC’s

Since the 1920’s, clinicians have noted an association between excessive alcohol ingestion and the development of infections. These observations suggest that alcohol interferes with the normal production and/or function of WBC’s, which form the body’s defense against microorganisms and other foreign substances. Because alcoholics commonly develop bacterial infections, much research has focused on alcohol’s effects on neutrophils, the primary cell of defense against bacterial invasion. However, alcohol also impairs the function of monocytes and macrophages, which attack bacteria and other microorganisms, and of lymphocytes, which mediate the immune response. Alcohol-induced impairment of neutrophils and monocytes is discussed in the following sections. (Alcohol’s effects on the immune system are reviewed in detail in the article by Szabo, pp. 30–41.)

### Neutrophils

When a severe bacterial infection occurs, the body’s response usually includes an increase in the number of WBC’s—especially neutrophils—in the blood, a condition called leukocytosis. In contrast, alcoholics suffering from bacterial infections often exhibit a reduced number of neutrophils in the blood (i.e., neutropenia). For example, in a study of 10 alcoholics with severe bacterial pneumonia or other bacterial infections, neutropenia was present in 5 patients when they were admitted to the hospital and developed in the other 5 patients within 24 to 48 hours (McFarland and Libre 1963). The neutropenia was transient, however, and in several patients a rebound leukocytosis occurred between 5 and 10 days after hospital admission.

The observed neutropenia may be related to impaired neutrophil development in the bone marrow. Thus, bone marrow analysis of alcoholic patients during the neutropenic stage demonstrated that virtually none of the neutrophil precursors had matured beyond an early developmental stage. Moreover, the neutrophil stores that are maintained in the bone marrow to allow a quick response to a bacterial infection were depleted more rapidly in active alcoholics than in healthy control subjects.

Alcohol consumption also interferes with the neutrophils’ ability to reach the site of an infection or inflammation (i.e., neutrophil delivery). When traveling to such a site, the neutrophils adhere to the walls of the blood vessels before migrating out of the blood vessels into the affected tissue. In tissue-culture experiments using nylon fibers to mimic this adherence, neutrophils could not adhere to the fibers if the blood samples were incubated with alcohol. This effect was more pronounced the higher the alcohol doses were. Neutrophils obtained from intoxicated volunteers had the same defect. The degree and duration of this adherence defect correlated with the inhibition of neutrophil delivery observed in the body. Moreover, drugs that corrected the adherence defect in tissue-culture experiments also improved neutrophil delivery in humans.

The function of neutrophils, including their adhesion ability, is regulated by hormonelike substances called leukotrienes. Thus, the impaired neutrophil functioning observed after alcohol treatment could be attributable to reduced leukotriene production or to the neutrophils’ inability to respond to the leukotrienes. Some research results indicate that alcohol can interfere with leukotriene production.

In an effort to overcome or prevent the alcohol-induced impairment of the body’s antibacterial defense, researchers have studied the effects of a growth factor called granulocyte-colony stimulating factor (G-CSF) in animal experiments. During normal neutrophil production in the bone marrow, G-CSF promotes the multiplication and functional activity of neutrophils. The studies found that G-CSF stimulated neutrophil recruitment specifically to the site of an infection and ameliorated the alcohol-induced impairment in the defense against bacterial infections.

### Monocytes and Macrophages

The monocyte-macrophage system, like neutrophils, constitutes an important line of defense against infections. Monocytes and macrophages clear invading microorganisms as well as foreign or defective proteins from the blood by engulfing and subsequently destroying them. Alcohol interferes with the function of the monocyte-macrophage system, with clinically significant consequences. For example, compared with healthy people, alcoholics are less resistant to infections by microorganisms that normally are eradicated by monocytes and macrophages, such as the bacteria that cause tuberculosis and various forms of pneumonia. Similarly, studies of intoxicated laboratory animals demonstrated reduced elimination of bacteria by the monocyte-macrophage system. These effects generally appear to be temporary. Thus, in alcoholic patients whose monocyte-dependent elimination of a defective form of albumin (a protein normally present in the blood) is reduced at admission to a hospital, monocyte function generally returns to normal within 1 week of abstinence from alcohol. Further studies indicate that alcohol impairs monocyte/macrophage function rather than production. Thus, the cells frequently remain at their normal locations in the tissues rather than migrate to the sites of infections. In addition, alcohol inhibits the monocytes’ adhesion abilities.

## Alcohol’s Effects on the Blood-Clotting System

Blood clotting, or coagulation, an important physiological process that ensures the integrity of the vascular system, involves the platelets, or thrombocytes,^4^ as well as several proteins dissolved in the plasma. When a blood vessel is injured, platelets are attracted to the site of the injury, where they aggregate to form a temporary plug. The platelets secrete several proteins (i.e., clotting factors) that—together with other proteins either secreted by surrounding tissue cells or present in the blood—initiate a chain of events that results in the formation of fibrin. Fibrin is a stringy protein that forms a tight mesh in the injured vessel; blood cells become trapped in this mesh, thereby plugging the wound. Fibrin clots, in turn, can be dissolved by a process that helps prevent the development of thrombosis (i.e., fibrinolysis).

Alcohol can interfere with these processes at several levels, causing, for example, abnormally low platelet numbers in the blood (i.e., thrombocytopenia), impaired platelet function (i.e., thrombocytopathy), and diminished fibrinolysis. These effects can have serious medical consequences, such as an increased risk for strokes.

### Thrombocytopenia

Thrombocytopenia is a frequent complication of alcoholism, affecting 3 to 43 percent of nonacutely ill, well-nourished alcoholics and 14 to 81 percent of acutely ill, hospitalized alcoholics. Thus, apart from acquired immune deficiency syndrome (AIDS), alcoholism probably is the leading cause of thrombocytopenia. Except for the most severe cases, however, the patients generally do not exhibit manifestations of excessive bleeding. Moreover, alcohol-related thrombocytopenia generally is transient, and platelet counts usually return to normal within 1 week of abstinence.^5^ Therefore, patients generally require no therapeutic intervention other than that needed to ease alcohol withdrawal. Only in patients whose thrombocytopenia is severe and associated with excessive bleeding are platelet transfusions indicated.

In many patients with thrombocytopenia, rebounding platelet numbers even exceed normal values. This rebound thrombocytosis after cessation of alcohol consumption also occurs in the majority of patients whose platelet counts are normal at the time of hospitalization. In these patients, the extent of the excess in circulating platelets usually is higher than in patients presenting with thrombocytopenia.

The exact mechanisms underlying alcohol-related thrombocytopenia remain unknown. Some researchers have suggested that alcohol intoxication itself, rather than alcohol-related nutritional deficiencies, causes the decrease in platelet numbers. This view is supported by findings that thrombocytopenia developed in healthy subjects who received a diet containing adequate protein and vitamin levels (including large doses of folic acid) and consumed the equivalent of 1.5 pints (i.e., 745 milliliters) of 86-proof whiskey for at least 10 days (Lindenbaum 1987). The subjects’ platelet levels returned to normal when alcohol consumption was discontinued. Similarly, platelet counts can be reduced in well-nourished alcoholics who do not suffer from folic acid deficiency. The available data also suggest that alcohol can interfere with a late stage of platelet production as well as shorten the life span of existing platelets.

Individual drinkers appear to differ in their susceptibility to alcohol-induced thrombocytopenia. Thus, clinicians have noted that some people who consume alcohol in excess repeatedly develop thrombocytopenia (often severely), whereas other drinkers maintain normal platelet levels.

In addition to differences in the quantity of alcohol consumed, inherited or acquired variations in an individual drinker’s biochemistry may account for these differences in susceptibility.

### Thrombocytopathy

Alcohol affects not only platelet production but also platelet function. Thus, patients who consume excessive amounts of alcohol can exhibit a wide spectrum of platelet abnormalities when admitted to a hospital. These abnormalities include impaired platelet aggregation, decreased secretion or activity of platelet-derived proteins involved in blood clotting, and prolongation of bleeding in the absence of thrombocytopenia.

Because alcohol impairs the function of the normal blood-clotting system, it also can adversely interact with over-the-counter and prescription medications that prolong bleeding or prevent coagulation. For example, alcohol can potentiate the prolongation of bleeding time caused by aspirin and other nonsteroidal anti-inflammatory drugs (NSAID’s) (e.g., ibuprofen or indomethacin), particularly when alcohol ingestion equivalent to about four drinks occurs simultaneously with or following ingestion of normal doses of these medications. As a result, the concomitant use of alcohol and aspirin or NSAID’s greatly increases the patient’s risk for gastrointestinal bleeding. Similarly, alcohol can enhance aspirin-induced fecal blood loss. To prevent such adverse reactions, health care professionals should proactively counsel patients who regularly consume alcohol about the proper choice and safe use of aspirin and other over-the-counter NSAID’s.

Alcohol also can interact with anticoagulants, prescription medications that prevent blood clotting and which are used to treat patients who are at increased risk of developing thrombosis or an embolism in the lung. One commonly used anticoagulant is warfarin. However, warfarin treatment is not indicated for alcoholic patients, because alcohol ingestion can significantly interfere with the proper management of warfarin maintenance therapy.

### Fibrinolysis

The body’s ability to prevent excessive bleeding using the coagulation system is balanced by the fibrinolytic system, which helps ensure blood flow in peripheral organs and tissues by dissolving inappropriate fibrin clots. Alcohol’s effect on fibrinolysis is controversial. Whereas some older studies reported an increase in fibrinolytic activity after alcohol consumption, more recent, better controlled studies have demonstrated that alcohol diminishes fibrinolysis the day after alcohol ingestion or during prolonged alcohol consumption. These observations suggest that alcoholics may be at increased risk for thrombosis.

Fibrinolysis is controlled in part by the presence in the blood of two proteins: tissue plasminogen activator (TPA) and plasminogen activator inhibitor 1 (PAI-1). TPA promotes fibrinolysis, whereas PAI-1 reduces fibrinolytic activity. The activities of both proteins must be well balanced to maintain an adequate level of fibrinolyis. Alcohol can alter the activities of both TPA and PAI-1. Thus, moderate alcohol consumption stimulates TPA activity. Because increased TPA activity reduces the risk of inappropriate blood-clot formation, this alteration may have a beneficial, cardioprotective effect. Conversely, recent studies also have indicated that high levels of alcohol or its degradation product, acetaldehyde, could stimulate PAI-1 production and thereby suppress fibrinolysis.

### Stroke

Alcohol-induced impairment of the blood-clotting and/or fibrinolytic systems can have serious medical consequences. Most significantly, clinical epidemiological data suggest that a recent bout of heavy drinking increases the drinker’s risk of suffering a hemorrhagic or ischemic stroke. During a hemorrhagic stroke, the blood flow to a brain area is impaired due to a ruptured blood vessel that results in bleeding in the brain. If the blood flow is interrupted because a blood vessel is blocked by a blood clot, the condition is called an ischemic stroke. Alcohol conceivably can contribute to both conditions by interfering with the normal coagulation system and by reducing fibrinolysis, respectively.^6^ For example, researchers have suggested that acetaldehyde interacts with some proteins of the blood-clotting system and thus induces abnormal coagulation. In addition to these direct effects on the blood components, alcohol may increase the risk of a stroke indirectly by altering the drinker’s blood pressure; heart rate; tone of the heart muscles; and “thickness,” or viscosity, of the blood. (For more information on alcohol’s effects on the cardiovascular system, see the article by Zakhari, pp. 21–29.)

## Summary

Numerous clinical observations support the notion that alcohol adversely affects the production and function of virtually all types of blood cells. Thus, alcohol is directly toxic to the bone marrow, which contains the precursors of all blood cells, as well as to the mature cells circulating in the bloodstream. Moreover, long-term excessive alcohol consumption can interfere with various physiological, biochemical, and metabolic processes involving the blood cells. The medical consequences of these adverse effects can be severe. They include anemia, which in severe cases can have debilitating effects; an increased risk of serious bacterial infections; and impaired blood clotting and fibrinolysis, which can cause excessive bleeding and place the drinker at increased risk of strokes. These direct effects may be exacerbated by the presence of other alcohol-related disorders, such as liver disease and nutritional deficiencies. Abstinence can reverse many of alcohol’s effects on hematopoiesis and blood cell functioning.

## Footnotes

1 In this article, the terms “chronic alcohol abuse” or “chronic excessive alcohol consumption” refer to the ingestion of 1 pint or more of 80- to 90-proof alcohol (i.e., about 11 drinks) per day. However, alcohol-related hematological problems can occur at much lower consumption levels. The drinker’s risk for developing these problems grows with increasing alcohol consumption.

2 For the definition of this and other technical terms used in this article, see the central glossary, pp. 93–96.

3 Less commonly, vacuole development in pronormoblasts also can occur after treatment with the antibiotic chloramphenicol. The two conditions can easily be distinguished, however, because in contrast to the alcohol-induced vacuolation, chloramphenicol-induced vacuolation is accompanied by the disappearance of virtually all later RBC precursors.

4 Platelets actually are not intact cells but disc-shaped cell fragments without nuclei that are released from giant precursor cells in the bone marrow called megakaryocytes.

5 Failure of the platelet counts to rise after 5 to 7 days of abstinence usually indicates the presence of another underlying disorder affecting the platelets.

6 Moderate drinking, however, has been shown to decrease the risk of ischemic stroke.
